# The Significance of Clinical Suspicion: From Pitting Edema to Streptococcus infantarius Endocarditis

**DOI:** 10.7759/cureus.72467

**Published:** 2024-10-27

**Authors:** Kevin T Dao, Jose Garcia-Corella, Breeanna Carlson, Hina Khanzada, Shravya Dharambhat, Arin Orogian, Kasey Fox

**Affiliations:** 1 Internal Medicine, University of California, Los Angeles (UCLA) - Kern Medical, Bakersfield, USA

**Keywords:** bacteremia, clinical suspicion, colon polyps, infective endocarditis, physical examination, streptococcus bovis, streptococcus infantarius, tubular adenomas

## Abstract

As advancements in medicine progress, greater emphasis has been placed on laboratory values and imaging rather than the clinical picture. This, as a result, has led to a decline in the use of clinical history and physical examinations. In many instances, some details of the history are glossed over, and performing a physical examination has become more lackadaisical. Here, we would like to present a unique case of a patient who presented to the emergency department with only pitting edema that was worsening for the past few weeks. Vitals were noted to be unremarkable, and the rest of the physical examination only revealed mild ascites and a new heart murmur, which was not present in his previous documented clinic visits. Due to this concerning physical examination finding, a cardiology and hepatology workup was done. It was then revealed that the patient had severe aortic and mitral valve vegetation suggesting infective endocarditis with severe aortic insufficiency due to *Streptococcus infantarius*. Due to the bacterium being a member of the *Streptococcus bovis* species, a colonoscopy was done, which revealed multiple tubular adenomas. A discussion regarding *S. infantarius* will be held, as well as the unique details of tubular adenomas causing *S. infantarius* bacteremia. The importance of physical examination and clinical suspicion will also be discussed.

## Introduction

*Streptococcus infantarius* is a gram-positive alpha hemolytic bacteria belonging to the* Streptococcus bovis* species, specifically *Streptococcus bovis* biotype II.1, along with *Streptococcus lutetiensis*. In most cases of *S. bovis-*related infective endocarditis, *Streptococcus bovis* biotype I, particularly *Streptococcus gallolyticus* bacteremia, resulted in approximately 43%-100% of cases developing infectious endocarditis [1]. With regard to *S. infantarius*, the cases of infective endocarditis were much less. A retrospective cohort study that included approximately 210 cases of *S. bovis* bacteremia estimated that of the *S. bovis* species, 16% were *S. infantarius* as opposed to 33% that were *S. gallolyticus* [2]. In most instances, patients with *S. bovis* infective endocarditis tend to portray symptoms of any other bacteremic endocarditis, such as fevers, fatigue, and night sweats [3]. However, once the bacterium is confirmed to be of the *S. bovis* species, many prior studies have highly recommended a colonoscopy due to the positive correlation between *S. bovis* infective endocarditis and colonic neoplasias [4-7]. In fact, one study noted that approximately 94% of patients who had *S. bovis* bacteremia were also noted to have colorectal cancer [8]. However, in the said study, the species of *S. bovis* varied but were predominately *S. gallolyticus*. Regardless, many patients who progress to *S. bovis* infective endocarditis tend to have various symptoms. Thus, there have not been many instances of patients who present with mild symptoms, although there have been cases of asymptomatic bacteremia with various bacterial species [9,10].

Here, we present a patient who arrives at the emergency department with complaints of bilateral pitting edema causing him mild foot pain but denies any other symptoms. The patient noted a significant alcohol history, and a physical examination revealed a new heart murmur. Due to concerns of new-onset heart and/or liver failure, further workup revealed that the patient not only had *Streptococcus infantarius* bacteremia but also infective endocarditis. The suspicion of underlying colonic pathology due to association with the bacterium was then confirmed by colonoscopy, which revealed various tubular adenomas. Here, we would like to detail the importance of physical examination and clinical indication since similar patients who are fairly asymptomatic could have significant underlying diseases, and a basic workup should be done to ensure patients with such diseases are managed accordingly.

## Case presentation

The patient is a 64-year-old man with a past medical history of alcohol dependence who presented to the emergency department with complaints of progressive bilateral pedal swelling for the past week as well as mild abdominal distension of unknown duration. The patient stated that he did not take any medication for his symptoms and noted that prior to this episode, he did not have any issues.

On physical examination, the vitals were within normal limits, the chest examination was unremarkable, and a cardiac examination revealed a grade 3/4 decrescendo diastolic murmur on the left lower sternal border and a grade 2/6 pansystolic murmur on the apex. No jugular venous distension was appreciated. Abdominal examination showed mild ascites, and 3+ pitting edema was noted in the bilateral lower extremity examination. The rest of the physical examination was unremarkable.

Due to this concerning finding of a new-onset murmur with bilateral lower extremity pitting edema and mild ascites, further workup was done. Blood work showed that the patient had an elevated white blood count with an increased neutrophil percentage and absolute neutrophil, as well as mildly elevated liver enzymes. However, the patient had no increase in band percentage. Bilirubin was also noted to be mildly elevated, as well as brain natriuretic peptide. The rest of the patient's blood work was noted to be unremarkable (Table 1). The patient was started on oral furosemide 40 mg twice daily and oral spironolactone 100 mg daily.

**Table 1:** Blood work on initial presentation WBC: white blood count, Hgb: hemoglobin, MCV: mean corpuscular value

Parameter	Value	Reference
Sodium	138 mmol/L	136-145 mmol/L
Potassium	3.8 mmol/L	3.5-5.1 mmol/L
Chloride	108 mmol/L	98-107 mmol/L
Calcium (corrected)	10 mg/dL	8.5-10.1 mg/dL
Magnesium	1.9 mg/dL	1.8-2.4 mg/dL
Phosphorus	3.8 mg/dL	2.5-4.9 mg/dL
Alanine transaminase	50 units/L	13-61 units/L
Aspartate transaminase	72 units/L	15-37 units/L
Direct bilirubin	1.2 mg/dL	0-0.2 mg/dL
Total bilirubin	2.6 mg/dL	0-1 mg/dL
Albumin	2.1 g/dL	3.4-5 g/dL
Erythrocyte sedimentation rate	83 mm/hour	<20 mm/hour
Brain natriuretic peptide	219 pg/mL	<65 pg/mL
WBC	22.5×10^3^/mcL	4.5-11×10^3^/mcL
Hgb	11.6 g/dL	13.2-17.4 g/dL
MCV	99.9 HI	80-98 HI
Platelet	91×10^3^/mcL	150-450×10^3^/mcL
Neutrophil %	81.9%	50%-75%
Lymphocyte %	6.3%	20%-45%
Bands %	3%	<12%
Monocyte %	11.5%	2%-12%
Eosinophil %	0%	<6%
Absolute neutrophil	18.4×10^3^/mcL	1.8-7.7×10^3^/mcL
Absolute lymphocyte	1.4×10^3^/mcL	1.2-4.5×10^3^/mcL
Absolute monocyte	2.6×10^3^/mcL	0.1-1×10^3^/mcL
Absolute eosinophil	0×10^3^/mcL	<0.7×10^3^/mcL
Hepatitis A antibody IgM	Nonreactive	-
Hepatitis B core antibody IgM	Nonreactive	-
Hepatitis B surface antigen	Nonreactive	-
Hepatitis C antibody	Nonreactive	-

**Figure 1:**
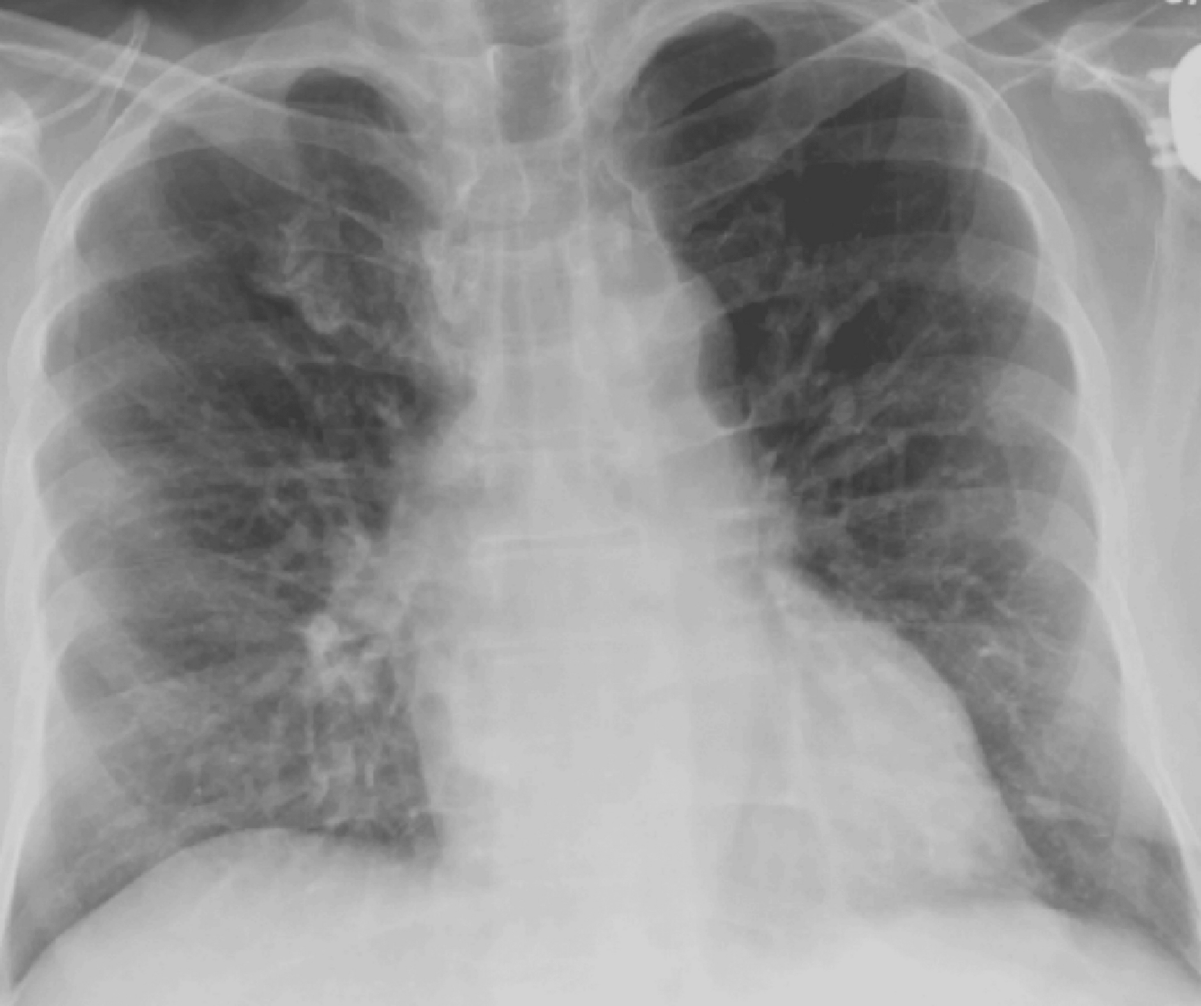
Chest X-ray Chest X-ray showing no acute cardiopulmonary process

**Figure 2:**
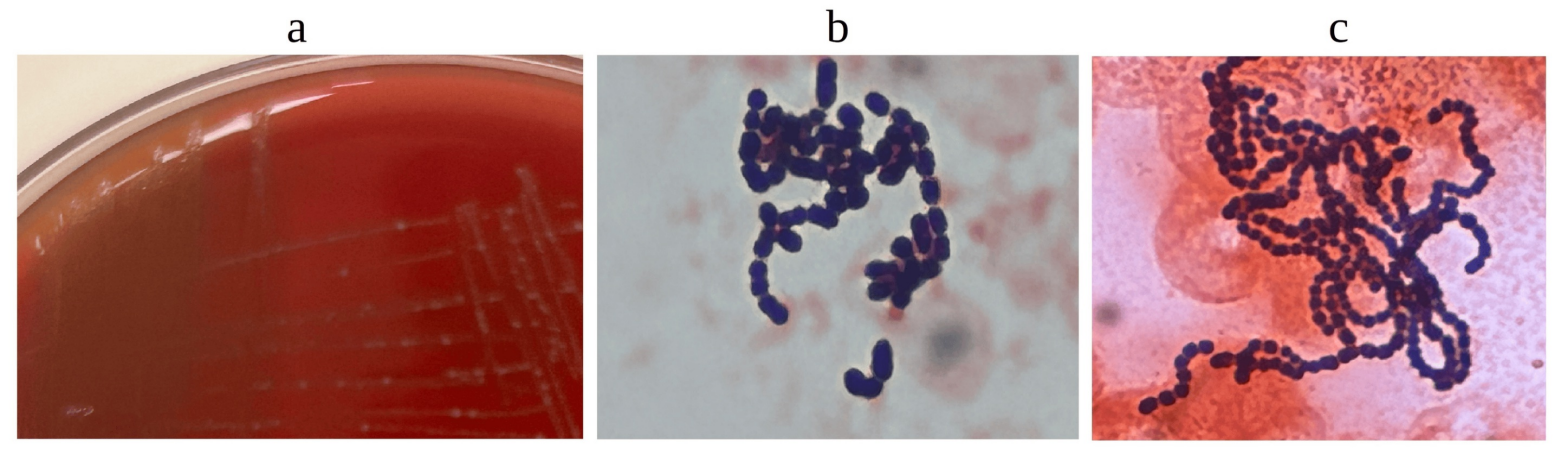
Blood cultures a: First blood cultures seen on blood agar showing alpha hemolytic *Streptococcus. *b: First set of blood cultures showing gram-positive cocci. c: Second set of blood cultures showing gram-positive cocci.

**Figure 3:**
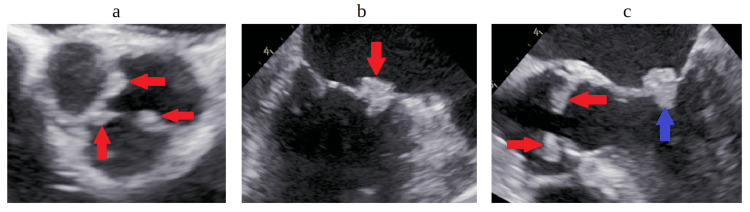
Transesophageal echocardiogram a: Short-axis view of the aortic valve showing vegetations highlighted by the red arrows. b: Mitral valve anterior leaflet showing vegetations highlighted by the red arrows. c: Five-chamber view with vegetations on the aortic valve showing vegetations highlighted by the red arrows and mitral valve showing vegetations highlighted by the blue arrow.

Blood cultures were done, as was a bedside transthoracic echocardiogram, which was noted to be unremarkable with an ejection fraction of >65%. A chest X-ray was done, which was unremarkable (Figure 1). An abdominal ultrasound was also done, which showed severe hepatocellular disease with multiple hepatic cysts, as well as small-volume ascites. Moreover, 2/2 blood cultures at two different body sites then resulted positive for alpha hemolytic *Streptococcus* (Figure 2a-2b). The patient was then started on 2 g of ceftriaxone, and repeat blood cultures were obtained. A formal transthoracic echocardiogram was repeated again, which showed a mobile echo density measuring approximately 0.9×0.7 cm seen on the aortic valve with increased velocity across the aortic valve and left ventricular outflow tract obstruction. The aortic valve deceleration time was 638.1 milliseconds with the aortic valve pressure half-time of 182 milliseconds indicating severe aortic root regurgitation with an ejection fraction of >65%. The pulmonic valve was difficult to visualize; however, the mitral and tricuspid valves showed only mild regurgitation but were otherwise unremarkable. Cardiology and infectious disease were both consulted. A second 2/2 bottle of repeat blood cultures after 48 hours of the first blood cultures was shown to grow positive for alpha hemolytic *Streptococcus* again (Figure 2c), and further speciation showed that the bacterium was *Streptococcus infantarius* belonging to the *Streptococcus bovis* group with sensitivities to ceftriaxone. A transesophageal echocardiogram was also ordered, which accurately portrayed multiple large mobile echoic densities on the left coronary cusp measured at 1.6×0.9 cm, the right coronary cusp measured at 1.3×1.0 cm, and the non-coronary cusp was measured at 1.1×0.8 cm, consistent with vegetations. A large mobile echo density measuring 1.1×1.3 cm was also attached to the anterior mitral valve leaflet close to the annular ring (Figure 3a-3c).

**Figure 4:**
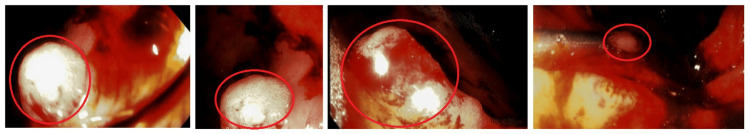
Colonoscopy Red circles showing the polyps noted on colonoscopy

Due to the correlation of the patient having *S. bovis* endocarditis and colon cancer, a colonoscopy was also done, which revealed a cecal polyp, ascending colon polyp, and transverse polyp, all of which were tubular adenomas (Figure 4). The patient was then started on six weeks of IV antibiotics with cardiology management. Throughout the hospital stay, the patient continued to remain asymptomatic, with only complaints of bilateral lower extremity pitting edema.

## Discussion

Generally, the majority of bacterial infections have been noted to be associated with carcinogenesis through the production of bacterial carcinogenic metabolites and/or chronic inflammation [11]. One study noted bacteria's ability to induce various mutations in the tumor suppressor genes, such as *TP53* [12]. Bacterial species in* S. bovis* were generally not a common cause of bacteremia and endocarditis. However, one study noted that *S. gallolyticus* encoded various proteins that allowed it to cause various pathologies such as colorectal cancer, bacteremia, and endocarditis [13]. Further studies later supported this association [2-7]. Despite this, there were also reports that members of the *S. bovis* genotype were also shown to have associations with colon polyps [14,15]. However, it is very rare for *Streptococcus bovis* biotype II, particularly *S. infantarius*, to cause bacteremia and infective endocarditis through colon polyps. However, in many instances, patients who progress to infective endocarditis with severe aortic valvular regurgitation tend to have more severe symptoms. In this case, the patient had only bilateral lower extremity swelling, with the physical examination revealing ascites, aortic diastolic heart murmur, and +3 bilateral pitting edema. This, as a result, should highlight the key importance of the patient's history and clinical examination.

In many situations, medicine has progressed to the point where certain clinical findings could provide crucial information beyond the patient's current state of health. In this instance, there was a high clinical suspicion that the patient had some form of underlying liver and/or heart disease. Due to the patient having a significant alcoholic history, further workup was warranted; however, due to the non-urgency of the matter, since the patient was noted to be hemodynamically stable, it was possible to have done such a workup in an outpatient setting. Such a setting would have been highly considered if patient blood work did not reveal an elevated white blood count (Table 1), which prompted blood cultures that had grown gram-positive alpha hemolytic *Streptococcus* (Figure 2a-2c). One study even discussed the importance of physical examination and how a patient who was incorrectly diagnosed with Bell's palsy due to an improper physical examination had acute embolic strokes secondary to infective endocarditis [16]. Another study also noted the unfortunate decline of physical examination due to increased dependence on medical technology [17]. Hopefully, this case details the importance of a detailed history, physical examination, and clinical suspicion since these skills have shown to be a crucial part of medical care.

## Conclusions

Overall, this case shows the importance of clinical suspicion as well as physical examination. If such a medical history and physical examination were neglected, then the patient could have been discharged from the emergency department and would likely further decompensate. An interesting element is that the patient was hemodynamically stable but had developed *S. infantarius* bacteremia leading to infective endocarditis through colon adenomas, as opposed to colon cancer, which is uncommon. Regardless, the key point of the case is to provide more awareness of the importance of the history of the present illness, clinical picture, and physical examination so that physicians will be able to catch more critical cases.
